# Childhood-Onset Movement Disorders Can Mask a Primary Immunodeficiency: 6 Cases of Classical Ataxia-Telangiectasia and Variant Forms

**DOI:** 10.3389/fimmu.2022.791522

**Published:** 2022-01-28

**Authors:** Geraldine Blanchard-Rohner, Anna Peirolo, Ludivine Coulon, Christian Korff, Judit Horvath, Pierre R. Burkhard, Fabienne Gumy-Pause, Emmanuelle Ranza, Peter Jandus, Harpreet Dibra, Alexander Malcolm R. Taylor, Joel Fluss

**Affiliations:** ^1^Paediatric Immunology and Vaccinology Unit, Division of General Pediatrics, Department of Pediatrics, Gynecology and Obstetrics, Geneva University Hospitals, University of Geneva, Geneva, Switzerland; ^2^Department of Clinical and Experimental Sciences, University of Brescia, ASST Spedali Civili, Brescia, Italy; ^3^Division of General Pediatrics, Department of Pediatrics, Gynecology and Obstetrics, Geneva University Hospitals, University of Geneva, Geneva, Switzerland; ^4^Pediatric Neurology Unit, Department of Pediatrics, Gynecology and Obstetrics, Geneva University Hospitals, University of Geneva, Geneva, Switzerland; ^5^Department of Neurology, University Hospitals of Geneva, Geneva, Switzerland; ^6^Division of Pediatric Oncology and Hematology, Department of Women, Child and Adolescent Medicine, Geneva University Hospitals, Geneva, Switzerland; ^7^CANSEARCH Research Platform for Pediatric Oncology and Hematology, Department of Pediatrics, Gynaecology and Obstetrics, Faculty of Medicine, University of Geneva, Geneva, Switzerland; ^8^Medigenome, Swiss Institute of Genomic Medicine, Geneva, Switzerland; ^9^Division of Immunology and Allergology, University Hospitals and Medical Faculty of Geneva, Geneva, Switzerland; ^10^Institute of Cancer and Genomic Sciences, University of Birmingham, Birmingham, United Kingdom

**Keywords:** ATM kinase activity, immunodeficiency, ataxia telangiectasia, movement disorder, cerebellar ataxia

## Abstract

Ataxia-telangiectasia (A-T) is a neurodegenerative and primary immunodeficiency disorder (PID) characterized by cerebellar ataxia, oculocutaneous telangiectasia, immunodeficiency, progressive respiratory failure, and an increased risk of malignancies. It demands specialized care tailored to the individual patient’s needs. Besides the classical ataxia-telangiectasia (classical A-T) phenotype, a variant phenotype (variant A-T) exists with partly overlapping but some distinctive disease characteristics. Here we present a case series of 6 patients with classical A-T and variant A-T, which illustrates the phenotypic variability of A-T that can present in childhood with prominent extrapyramidal features, with or without cerebellar ataxia. We report the clinical data, together with a detailed genotype description, immunological analyses, and related expression of the ATM protein. We show that the presence of some residual ATM kinase activity leads to the clinical phenotype variant A-T that differs from the classical A-T. Our data illustrate that the diagnosis of the variant form of A-T can be delayed and difficult, while early recognition of the variant form as well as the classical A-T is a prerequisite for providing a correct prognosis and appropriate rehabilitation and support, including the avoidance of diagnostic X-ray procedures, given the increased risk of malignancies and the higher risk for side effects of subsequent cancer treatment.

## Introduction

Ataxia-telangiectasia (A-T) is a rare neurodegenerative and immunodeficiency disorder caused by biallelic pathogenic variants in the *ATM* gene. It belongs to the group of genome instability syndromes that, like A-T, show an unusual sensitivity to ionising radiation and a cancer susceptibility. Its onset is often seen in infancy with cerebellar ataxia being the principal feature (1). Ocular telangiectasias develop later but are usually apparent by age 10 years. Due to the progressive course of the disorder, a wheelchair is usually required for mobility by early teen age (2). Clinical suspicion can be confirmed by measuring the serum alpha-foetoprotein (AFP), which is an easily detectable and reliable biological hallmark of the disorder.

Despite these well-known characteristics, early diagnosis of A-T might be challenging. Indeed, the abovementioned cardinal clinical features might be only partially present depending primarily on the particular type of identified *ATM* mutations and the amount of residual ATM kinase activity. The current expansion of the original phenotype is also closely linked with the ongoing discoveries of the multiple and versatile roles of the ATM protein that go beyond its critical role in maintaining the genomic integrity (3, 4). Indeed, ATM is also important in cell homeostasis, synaptic trafficking and early neurodevelopment by promoting neurogenesis and migration. The neurodegenerative features in adult neurons reflect the DNA damage during oxidative stress, which progresses with age (3–5).

Numerous studies have shown that the expression of either a low level of normal ATM, arising from a leaky splice site mutation, or the presence of some mutant ATM arising from a missense mutation, which are both associated with retention of some kinase activity/signalling, often results in a distinct neurological clinical phenotype compared with the biallelic ATM null patients (6–8). Therefore, the name A-T can be misleading as both ataxia and telangiectasia can be absent in some patients retaining a low level of ATM kinase activity. In those patients, designated AT variants, the neurological presentation does not necessarily include cerebellar features at the forefront and might be essentially extrapyramidal (i.e., dystonia, choreoathetosis, resting tremor, parkinsonism and myoclonus) or mixed, with little to no systemic features and no immunological impairment (6, 9–11).

In addition, Micol et al. showed that the clinical outcome, particularly the risk of cancer, was more severe in those A-T patients with biallelic null mutations resulting in loss of expression of all ATM compared with those with hypomorphic mutations who were more prone to respiratory tract infections (12). Despite these distinctive features, the risk of malignancy is significantly increased in both types of A-T, with common haematological involvement in the first two decades of life and increased risk of solid organ malignancies during young adulthood (13, 14), making crucial an early recognition that will enable proper management and follow-up (14, 15).

We share here our experience with 6 patients that illustrate well the various phenotypic presentations of A-T and highlight the distinctive course of patient with the AT-variant phenotype, which might be identified only in young adulthood. The clinical description goes along a detailed genotype description and related expression of the ATM protein.

## Patients and Methods

### Probands

Clinical data were retrospectively collected from case notes for all individuals with classical and variant A-T who have attended the paediatric immunology and neurology units of the University Hospitals of Geneva (HUG). Analysis included cross-sectional data using the clinical assessment recorded at the diagnosis (T0) and the most recent follow-up visit (T1).

### Neurological Assessment

Clinical neurological assessment was performed by a paediatric neurologist with a special interest in A-T. An overall evaluation of disease severity was made using an assessment of movement disorders and the scoring system of The Scale for Assessment and Rating of Ataxia (SARA) and/or International Cooperative Ataxia Rating Scale (ICARS) scores. The maximum score for SARA is 40, while for ICARS it is 100 (16).

### Immunological Assessment

Immunological investigations included immunoglobulin levels (immunoglobulin IgG, IgA, IgM), AFP serum levels, lymphocyte subset analysis, and assessment of serum vaccine antibody to pneumococci, tetanus and haemophilus influenza type b. History of immunizations, infections and treatment was recorded.

### Molecular Genetic Studies

Patients 1, 2, 3, 5 and 6 had exome sequencing with targeted bioinformatics analysis either on *ATM* or on a panel of genes implicated in neurological disorders. Patient 4 had targeted analysis using PCR and Sanger sequencing.

### ATM Activity Signalling

A lymphoblastoid cell line (LCL) was derived from each patient’s blood. Immunoblotting for ATM expression and ATM activity assays were performed using methods previously described (7, 8). Briefly, following activation of ATM by exposure of cells to 2-Gy x-rays, cells were harvested at 30 min and the ATM present in patient derived cells was tested for its ability to phosphorylate a group of target proteins (Smc1, KAP1, Nbn and CREB) using phosphospecific antibodies. Antibodies used for immunoblotting were ATM 11G12 (custom made mouse monoclonal), pATM Ser1981 #AF1655 (R&D Systems, Abingdon, UK), Smc1 #A300-055A, pSmc1 Ser966 #A300-050A, KAP-1 #A300-275A, pKAP-1 Ser824 #A300-767A (Bethyl Laboratories, Universal Biologicals, Cambridge, UK), Nbs1 #Ab23996, pNbs1 Ser343 #Ab47272, CREB #Ab32515 (Abcam, Cambridge, UK), pCREB Ser121 #NB100-410 (Novus Biologicals, Cambridge, UK).

**Figure 1:**
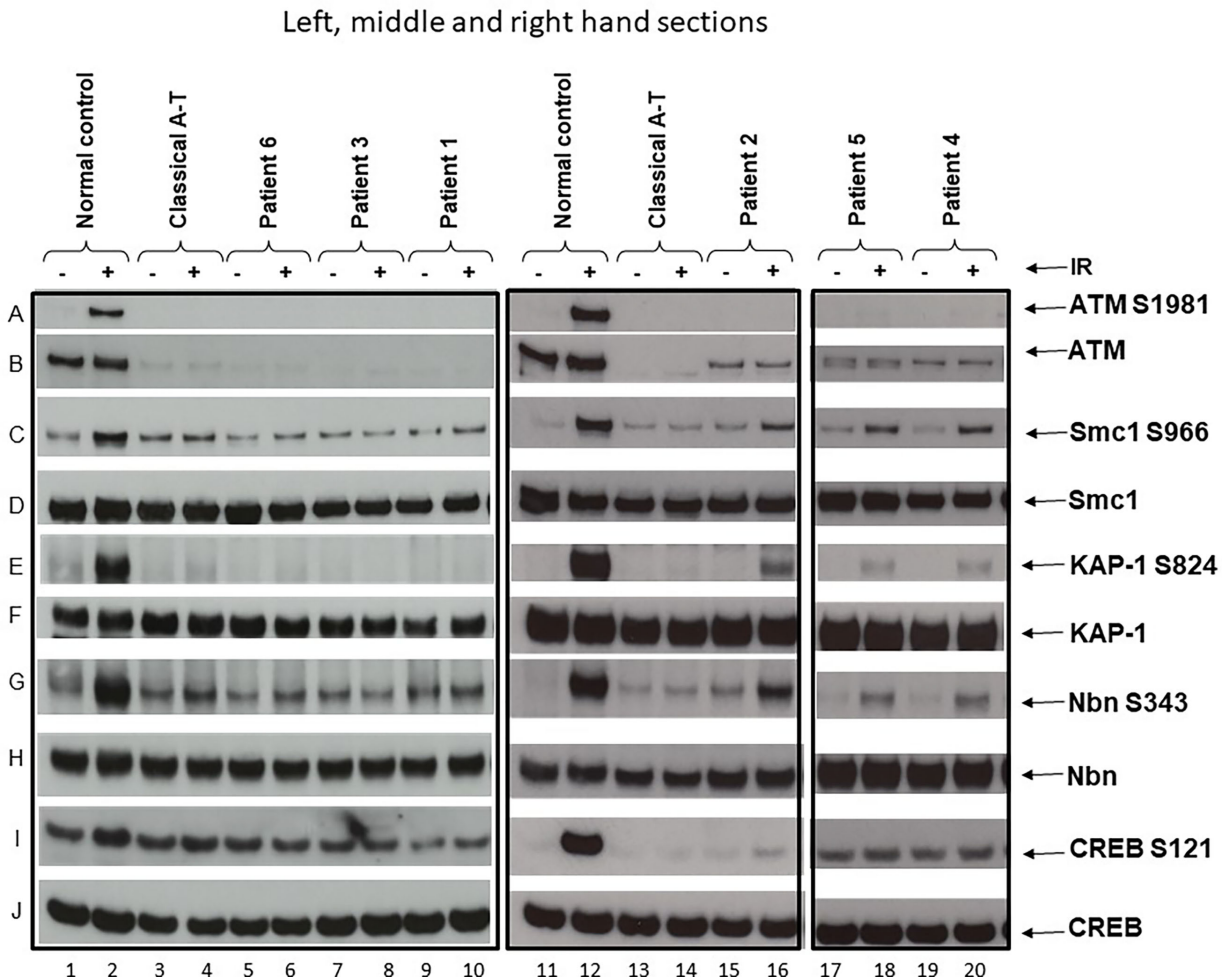
ATM and ATM kinase activity/signalling in cells from ataxia telangiectasia patients 1–6. Assay showing the presence or absence of ATM in cells from each patient and whether this was associated with some activity/signalling ability. Cells were either irradiated (IR) with 2-Gy x-rays or not irradiated. The left-hand section show the results of the three classical patients, the middle section the results of variant patient 2, and the right-hand section the results of variants 4 and 5. Left-hand section, lanes 1 & 2—positive control. **(A)** Cells from a normal individual showing presence of ATM protein **(B)** and normal ATM activity/signalling showing phosphorylation of the targets SMC1 ser966 **(C)**, KAP-1 ser 824 **(E)**, Nbn ser343 **(G)**, and CREB ser121 **(I)**. There is a strong signal for each of these in lane 2 after activation. The total levels of SMC1, KAP-1, Nbn, and CREB are also shown in **(D, F, H, J)**, respectively. Lanes 3 and 4—negative control. Cell lysate from a classical A-T patient with mutations ATM, c.1355delC; p.(Thr452AsnfsTer21) and c.3802delG; p.(Val1268Ter). Both are frameshift mutations leading to instability and loss of the ATM from both alleles. There is no differential phosphorylation of these targets in this patient’s cells inane 4—consistent with the patient having A-T; there is no ATM **(B)**. Lanes 5 and 6 show cell lysates from patient 6, lanes 7 and 8 lysates from patient 3, and lanes 9 and 10 lysates from patient 1. Just as with the negative control (lane 4), lysates from these three patients show absence of ATM protein **(B)** and absence of a differentially increased phosphorylation induced by irradiation, as indicated by absence of phosphorylation of SMC1 ser966, KAP-1 ser824, Nbn ser343, and CREB ser121 in lanes 6, 8, and 10, respectively. Middle section: lanes 11 and 12 show lysates from the same normal control as in lanes 1 and 2. Lanes 13 and 14 are cell lysates from the same classical A-T patient as in lanes 3 and 4. Lanes 15 and 16 are lysates from variant patient 2. **(B)** confirms that the lysate shows a low level of ATM protein. In contrast with the negative control (lane 14), this lysate shows a clear low level of ATM kinase activity/signalling as indicated by the presence of some moderate phosphorylation of SMC1 ser966, KAP-1 ser824, Nbn ser343, or CREB ser121 in lane 16. Right-hand section, lanes 17 and 18 and 19 and 20 are lysates from variant patients 4 and 5. The positive and negative controls have been removed but were the same as in lanes 1–4 and 11–14. **(B)** confirms that the variant lysates show a low level of ATM protein. In contrast to the negative controls in lanes 4 and 14, these lysates also show a clear low level of ATM that has some retained kinase activity/signalling as indicated by the presence of moderate phosphorylation of SMC1 ser966, KAP-1 ser824, and Nbn ser343 in particular.

An LCL derived from a normal individual and another derived from a known classical A-T patient were used as positive and negative controls for ATM activity/signalling. Patients were classified as having classical or variant A-T based on either the absence of any detectable activity/signalling or the presence of some residual ATM activity/signalling, respectively (**Figure 1**).

### Case Description

Patient 1 (**Table 1**) is a 7-year-old boy born from non-consanguineous parents of European ancestry. He was first seen at our developmental clinic at 19 months of age, because his motor milestones were delayed while his cognitive function was preserved. He sat at 6 months and started standing and shuffling at 7–8 months. Despite his initial progress, he did not achieve independent walking before 18 months and started falling a month later. He also demonstrated difficulty in drinking and had started to drool. The parents also noticed bizarre posturing of the arms and hands. He was referred to the neurology outpatient clinic at 20 months. Brain MRI was unremarkable, and a primary dystonia was first suspected. A few months later, mild hand tremor along with an ataxic gait led however to a broader workup. The finding of extremely low serum IgG in association with elevated AFP (89.7 mcg/l) raised the suspicion of A-T. This was confirmed by genetic analysis revealing compound heterozygosity for likely pathogenic and pathogenic variants in the *ATM* gene (NM_000051.3), c.2455T>C;(p.Cys819Arg), and c.8264_8268del;(p.Tyr2755CysfsTer12), respectively. Each parent is heterozygous for one of the variants identified. A lymphoblastoid cell line showed no detectable ATM protein and therefore no kinase activity (**Figure 1**, lane 6). The immunologic workup revealed a severe immunodeficiency resulting in an almost complete absence of IgG and IgA and decreased T and B cells, but normal T cell lymphocyte function with anti-CD3 and tetanus toxoid. There was no history of recurrent and frequent infections. At the age of 2 years, he started on monthly intravenous immunoglobulin substitution at 2 years old and later switched to subcutaneous immunoglobulins. He was initially treated with prophylaxis cotrimoxazole, which was later discontinued because of profound neutropenia. Every winter, he could not attend the day care program because of recurrent upper respiratory viral infections, with difficulty to regain weight. So far, he has not suffered any severe infection. At present, he walks independently for short distance, despite persisting ataxia and dystonic posturing. His disease is globally stable, and he continues to attend mainstream school with additional care. Concerning the nutritional aspect, he was put on enteral nocturnal feeding at 4 years because of failure to thrive due to poor appetite and caloric intake. He also suffers from chronic constipation requiring daily laxatives. SARA and ICARS were not performed at diagnosis due to an age limit. During the last follow-up visit, both scores were 14 and 41, respectively.

**Table 1:** Clinical, genetic and biological characteristics of the 6 patients.

Patient	Patient 1:		Patient 3:		Patient 6:		Patient 2 :		Patient 4:		Patient 5:	
	Classical A-T		Classical A-T		Classical A-T		Variant AT		Variant AT		Variant AT	
Current age	7y		10y		4y		29y		25y		14y	
Age at diagnosis	19mo		9y		20mo		23y		24y		12y	
ATM mutation (NM_000051.3) Variant 1	c.2455T>C; (p.Cys819Arg) c.8264_8268del; (p.Tyr2755CysfsTer12)		c.7517_7520delGAGA; (p.Arg2506ThrfsTer3) c.8264_8268delATAAG; (p.Tyr2755CysfsTer12)		c.790delT; (p.Tyr264IlefsTer12) c.103C>T; (p.Arg35Ter).		c.6047A>G; (p.Asp2016Gly) c.6385T>G; (p.Tyr2129Asp)		c.6154G>A; (p.Glu2052Lys c.6355delG; (p.Val2119Ter)		c.6154G>A; (p.Glu2052Lys c.6355delG; (p.Val2119Ter)	
Variant 2												
Patient	Patient 1:		Patient 3:		Patient 6:		Patient 2 :		Patient 4:		Patient 5:	
	**Classical A-T**		**Classical A-T**		**Classical A-T**		**Variant AT**		**Variant AT**		**Variant AT**	
Neurological assessment (Age at onset)*	Ataxia (18mo) Bradykinesia (18mo)		Ataxia (5y) Choreo-athetotic movements (6y) Oculomotor apraxia (9y) Axonal neuropathy (9y) Bradykinesia (9y)		Ataxia (11mo), dystonia (20mo), Dysarthria and drooling (3y)		Dysarthria (2-3y) Axial myoclonus (1-2 y)		Dysarthria (2y) Instability of independent walk (2-3y) Arms tremor, dystonia and choreo- athetotic		Instability of independent walk (7y)	
	Dystonia (19mo) Instability of independent walk (5y)						Mild axial and limb dystonia (16y)		movements		Dystonia and choreo-athetotic movements (12y)	
							Resting tremor					
							(16y)					
							Mild axonal					
SARA T0^a^	NA		13,5		NA		4		4		0	
T1^a^	14		21		18,5		3		4		1	
ICARS T0^a^	NA		35		NA		11		7		1	
T1^a^	41		52		50		10		8		2	
Patient	Patient 1 (at 9mo): Classical A-T		Patient 3 (at 10y): Classical A-T		Patient 6 (at 20mo): Classical A-T		Patient 2 (at 25y): Variant AT		Patient 4 (at 24y): Variant AT		Patient 5 (at 13y): Variant AT	
**Ig levels (g/l):**	**0.07**	N.V.*	7.86	N.V.*	5.62	N.V.*	**3.34**	N.V.*	10.7	N.V.*	11.7	N.V.*
IgG IgA	**0**	3.7-11.7	**0**	6.5-14.6	0.3	3.8-17.9	**0.16**	16-Jul	1.13	16-Jul	2.05	6.5-14.6
IgM	1.03	0.17-1.32	**2.4**	0.7-4	0.8	0.17-1.32	**0.24**	0.7-4	0.63	0.7-4	1.08	0.7-4
		0.31-1.34		0.4-2.3		0.31-1.34		0.4-2.3		0.4-2.3		0.4-2.3
**Vaccine-Abs** Tetanus (UI/l) Diphtheria (UI/l) Measles (UI/l) Varicella (UI/l) *S.pneumoniae* (mg/l)	NA^ NA NA NA NA		N (1758) N (1079) N (648) N (702)		N (2194) N (446) NA		N (2496) N (316) N (200) N (227) **All neg**		N (1088) N (377) NA		N (2250) N (1001) NA	
			N (4/7 ≥0.5) ~		**No protection (<50) No protection (2/7)** ~				N (254) N (4/7) ~		N (1542) N (7/7) ~	
**Lymphocyte Prolif**.	N N NA NA		N N NA NA		N NA NA NA		N		N		N	
Anti-CD3							**No response**		**No response**		**No response**	
Tetanus							NA NA		N		N	
*Candida albicans*									**No response**		**No response**	
*S.pneumoniae*												
	Patient 1 (at 19mo)		Patient 3 (at 10y)		Patient 6 (at 20mo)		Patient 2 (at 25y)		Patient 4 (at 24y)		Patient 5 (at 13y)	
	**Classical A-T**		**Classical A-T**		**Classical A-T**							
**Lymphocyte Typing** Lymphocytes T cells	Cell/mm	Cell/mm3	Cell/mm	Cell/mm3	Cell/mm	Cell/mm3	Cell/mm	Cell/mm3	Cell/mm	Cell/mm3	Cell/mm	Cell/mm3
CD4	3	1700-6900	3	1000-5300	3	1700-6900	3	1140-3380	3	1140-3380	3	1000-5300
CD8	**838**	780-2240	**1071**	780-2240	**1580**	900-4500	1786	780-2240	1913	780-2240	1166	800-3500
NK	**455**	490-1640	**649**	490-1640	**821**	500-2400	1164	490-1640	1352	490-1640	**790**	400-2100
B cells CD27+, IgD+ CD27+, IgD- CD38higIgMhi (transit) CD21low CD38low	**295**	170-880	**290**	170-880	**370**	300-1600	570	170-880	946	170-880	517	200-1200
	**95**	80-690	**67**	80-690	388	100-1000	370	80-690	293	80-690	208	70-1200
	173	200-2100	166	200-600	526	200-2100	**40**	80-490	69	80-490	77	200-600
	**132**	20-180	**113**	20-70	**116**	20-180	299	Oct-80	416	Oct-80	214	20-70
	**1**	20-220	**16**	30-110	**12**	20-220	**159**	20-90	63	20-90	**16**	30-110
	27	20-200	41	Oct-60	**16**	5.7-28	42	0-30	81	1.6-30	**18**	11-111
	**0**	Oct-60	**0**	30-Oct	**2**	Oct-60	0	20-Oct	9	20-Oct	19	30-Oct
	16		28		10			26	43		14	
	Patient 1 (at 19mo) Classical AT		Patient 3 (at 10y) Classical AT		Patient 6 (at 20mo) Classical AT		Patient 2 (at 25y) Variant AT		Patient 4 (at 24y) Variant AT		Patient 5 (at 13y) Variant AT	
Alpha fetoprotein	89.7		520-580		134		78		154		58.1	
(mcg/l)												
Other	Failure to thrive		Supraventricular tachycardia		Chronic constipation		Diabetes type 1		Scoliosis, chronic nasal obstruction		Chronic constipation, Oral allergy syndrome and allergic rhinoconjunctivitis	
Support/ Treatment	IVIG/SC Ig Gastrostomy Ergotherapy Physiotherapy, Speech therapy		Ergotherapy, Physiotherapy, Speech therapy		Osmotic laxatives (Macrogol), Speech and physio-therapy		Insulin		Antiparkinsonian agents (Levodopa+benserazi de), Physiotherapy, Septoplasty and turbinoplasty (2018)		Osmotic laxatives (Macrogol), Antihistamines symptomatic treatments	

*Age at onset years (y) or months (mo).

a T0 neurological assessment at diagnosis.

b T1 neurological assessment at current age.

^*^N.V. normal values for age.

^^^NA not applicable.

^~^Number of serotypes with protective antibodies (≥0.5 mg/l).

N normal (protective).

Values of lymphocyte subsets and immunoglobulin levels marked in boldface are less than the age-related normal value. For normal values of lymphocyte subsets, see (17).

**Patient 2** (**Table 1**) is a 29-year-old male born to non-consanguineous Kurdish parents. He was known with a long history of non-progressive movement disorders since early childhood in the setting of preserved intellectual abilities. During adolescence, the combination of mild dystonia and axial myoclonus of the upper trunk led first to consideration of the diagnosis of myoclonus dystonia syndrome, which could not be genetically explored at that time. During a visit at the adult movement disorders clinic at the age of 22 years, a new onset of mild resting and intentional tremor in association with mild parkinsonian features prompted to repeat a brain MRI as well as a DaTscan in order to detect any abnormalities within the nigrostriatal pathways. Structural brain imaging revealed a global cerebellar atrophy that, in retrospect, was present to a much lesser degree 10 years earlier and had been overlooked. The DaTscan was fully normal. In addition, a chronic axonal sensorimotor neuropathy was demonstrated on electrophysiological studies in spite of minimal peripheral nervous system manifestations. Exome sequencing with targeted bioinformatics analysis on a panel of 1,400 genes implicated in various neurological disorders showed a compound heterozygosity for two likely pathogenic variants in the *ATM* gene (NM_000051.3), c.6047A>G;(p.Asp2016Gly) and c.6385T>G;(p.Tyr2129Asp). Each parent is heterozygous for one of the variants identified. The AFP level was at 78 mcg/l. Functional studies of the ATM kinase protein showed a low level of ATM which also retained a reduced level of ATM kinase activity/signalling (**Figure 1**, lane 12). The immunological workup revealed low IgG/IgA/IgM, despite normal vaccine antibody, lymphocyte subpopulation, and function. His infectious history was unremarkable. He developed a Type I diabetes at 27 years of age. At the last visit, in August 2020, he had dysarthria, mild axial dystonia, some choreic movements, resting tremor of the right upper limb and minimal ataxia only apparent at tandem gait. He is still walking normally. He is now undertaking postgraduate studies in a foreign country. A variant form of A-T was retained as the likely diagnosis. SARA and ICARS at the time of diagnosis were 3 and 4 respectively, while they were 10 and 11 respectively at the last follow-up visit.

**Patient 3** (**Table 1**) is a 10-year-old female, born from non-consanguineous parents of European ancestry and followed since infancy for supraventricular tachycardia. From the age of 5, subtle gait instability was noted. Between 6 and 8 years, she presented worsening static ataxia, dysmetria, occasional myoclonic jerks and choreoathetosis movements of the left upper limb. She was referred to a paediatric neurologist, and on examination, she also exhibited ocular telangiectasia and oculomotor apraxia. Her AFP level was increased at 520 mcg/l. A-T was strongly suspected and confirmed genetically; there was compound heterozygosity for two pathogenic variants in the *ATM* gene (NM_000051.3), c.7517_7520delGAGA;(p.Arg2506ThrfsTer3) and c.8264_8268delATAAG;(p.Tyr2755CysfsTer12) respectively. Each parent is heterozygous for one of the variants identified. Her cell lysate showed absence of ATM protein and activity (**Figure 1**, lane 8). Currently, she suffers from increasing walking difficulty because of gait ataxia and involuntary movements and also exhibits mild dysarthria, yet her speech remains intelligible. She continues to attend mainstream school with additional care: special needs assistants (SNA) and assistive technology. The immunological workup revealed normal IgG and IgM but absent IgA, normal vaccine antibody, low B and T cell subpopulations but normal lymphocyte function and an unremarkable infectious history. SARA and ICARS at diagnosis were 13.5 and 35 respectively, while during the last follow-up they reached 22 and 57 respectively.

**Patient 4** (**Table 1**) is a 25-year-old man born from non-consanguineous Kurdish parents, known since the age of 2 years for non-progressive gait instability, gross motor impairment and dysarthria. An initial brain MRI performed at the onset of symptoms at an outside institution was reported as normal. At 11 years, his gait was surprisingly normal, but a mild truncal instability was observed while sitting, and continuous choreic movements were noted. A repeat brain MRI and a lumbar puncture were normal. At 15 years, the gait instability had reappeared, and occasional paroxysmal dystonic postures predominant on the left side were noted in addition to the abovementioned choreic movements. Genetic investigations were performed at 24 years, following the diagnosis in his sister (patient 5); compound heterozygosity for a likely pathogenic and a pathogenic variant (NM_000051.3), c.6154G>A;(p.Glu2052Lys) and c.6355delG;(p.Val2119Ter) respectively was confirmed in the *ATM* gene. Each parent is heterozygous for one of the variants identified. The AFP level was at 154 mcg/l. Functional studies showed that there was a reduced level of ATM protein in the lymphoblastoid cell line and also a low level of ATM activity (**Figure 1**, lane 14). At the last follow-up visit, 1 year later, all abnormal movements had improved under levodopa. The immunological workup revealed normal serum immunoglobulin levels, and mitogen-stimulated lymphocyte and T-cell and B-cell counts were normal whereas NK-cells were reduced. At diagnosis, SARA and ICARS were 5 and 15 respectively, while during the last follow-up visit they were 4 and 14 respectively. He is still walking normally.

**Patient 5** (**Table 1**), the sister of patient 4, is a 14-year-old girl, known for dystonia, choreic movements and fatigability first observed in early childhood. These were all reported as much milder than those of her brother and did not prompt the parents to seek medical advice until investigations were performed on her brother. At this time (6 years of age), no clinical symptoms were observed that supported a diagnosis of A-T. At 12 years, the patient complained of an exercise-induced dystonic posture involving the right ankle appearing after 10 min of motor activity and a sustained extension of her right 5th digit when playing the flute. Genetic investigations performed at this age revealed the same variants as her brother: i.e. compound heterozygosity for likely pathogenic and pathogenic variants in the *ATM* gene (NM_000051.3), c.6154G>A;(p.Glu2052Lys) and c.6355delG;(p.Val2119Ter) and also confirmed to cause a reduced level of ATM protein and low level ATM activity (**Figure 1**, lane 16). Each parent is heterozygous for one of the variants identified. The AFP level was at 58.1 mcg/l. The immunological workup showed normal T cell subset counts and the mitogen-stimulated lymphocyte proliferation. The low levels of CD27IgD +/- cells were associated with normal B cell counts and serum immunoglobulin levels in the normal range. At diagnosis, SARA and ICARS were 0 and 1 respectively, while at the last follow-up visit, they were 2 and 3 respectively. She is still walking normally. She attends mainstream school and has very good grades.

**Patient 6** (**Table 1**) is a 3-year-old boy born after an uneventful pregnancy from non-consanguineous parents of European ancestry. He was evaluated by a paediatric neurologist at 20 months because of slowing acquisition of motor milestones and motor abnormalities, while cognitive development was maintained. He walked independently at 11 months, but his gait failed to improve during his second year and he remained unsteady with frequent falls.

He was primarily referred to a paediatric otolaryngologist because a vestibular dysfunction was first suspected, but a few months later the ataxic gait led to a neurological workup. On clinical examination, he exhibited intermittent dystonic postures of the extremities, gait ataxia and mild dysarthria. Cognitive development was normal. No ocular telangiectasia and oculomotor apraxia were noted. An elevated AFP serum level (134 mcg/l) was identified. The clinical suspicion of A-T was confirmed by genetic analysis revealing a compound heterozygosity for two pathogenic and likely loss-of-function variants in the *ATM* gene (NM_000051.3), c.790delT;(p.Tyr264IlefsTer12) and c.103C>T;(p.Arg35Ter). Each parent is heterozygous for one of the variants identified. Functional studies of the ATM kinase protein showed absence of detectable ATM protein and also no activity/signalling (**Figure 1**, lane 10). The immunological workup showed decreased B and T cell subset counts but normal mitogen-stimulated lymphocyte proliferation as well as immunoglobulin serum levels. His history of infections was unremarkable. Currently, he suffers from drooling, and his walking is difficult because of increasing ataxia and involuntary movements. SARA and ICARS were not performed at diagnosis due to an age limit. During the last-follow-up, they were 18.5 and 50 respectively.

## Discussion

In recent years, it has become increasingly clear that the clinical phenotype of A-T varies from the severe classical phenotype, including early-onset cerebellar ataxia (cerebellar symptoms) to a disorder with milder neurological impairment with later onset and variable forms of movement disorders (13). It has been postulated that the presence of some residual ATM kinase activity may “protect” the patient from the “classical” disease progression (8). The six patients described here illustrate the phenotypic variability of A-T that can present in childhood with prominent extrapyramidal features, with or without cerebellar ataxia. Pure extrapyramidal symptoms, which are uncommon in the course of classical A-T, suggest possible differences in neuronal vulnerability and pathway involvement between the classical and variant forms of the disease (8). Cognitive development appears to be preserved in both forms of the disease.

In our case series, cerebellar ataxia was absent in patients 2, 4 and 5 in whom a residual ATM kinase activity was detected. This was likely to have been derived from the c.6385T>G;(p.Tyr2129Asp) mutant protein in patient 2 as the second mutant protein c.6047A>G p.(Asp2016Gly) has been observed in another A-T patient who do not have any ATM activity/signalling (not shown). In patients 4 and 5, it is the mutant protein c.6154G>A;(p.Glu2052Lys) that provides the activity/signalling. While there is a clear increased phosphorylation of three targets (Smc1 ser966, KAP-1 ser824 and Nbn ser343, the increase is less obvious in this instance for CREBser121, hence the reason for using several targets. These are both mutations that can plausibly be associated with some expression of ATM and some retained kinase activity/signalling. These cases were diagnosed in young adulthood as their phenotype was much milder than the others. Moreover, they show a much more indolent course with minimal progression over two decades (18). These cases fit the clinical–biological picture of variant A-T and highlight the possibility that the more residual ATM kinase activity is present, the milder the neurological features may be, often with prominent extrapyramidal features and longer preserved ambulation (7, 8). The presence of residual ATM kinase activity is also associated with mild axonal polyneuropathy, late-onset anterior horn cell degeneration and adult-onset oculomotor apraxia that can mimic other neurological disorders (11, 14). In addition, patients with the A-T variant might not exhibit oculomotor and bulbar signs (11). *ATM* pathogenic variants, mainly truncating or severe missense mutations, found in patients 1 (missense p.Cys819Arg), 3 and 6 respectively resulted in complete absence of ATM protein and thus no kinase activity. The history of infections of these patients was unremarkable although some had immunological impairment, such as lymphopenia, hypogammaglobulinemia and in one of them even the absence of B cells and very low T cells (**Table 1**). These three patients were all diagnosed in early childhood because of movement disorders with marked ataxia, variable extrapyramidal features and rapid deterioration of neurological impairment. Although the cerebellar and neurological degeneration progresses in classical A-T, it has been suggested that immune deficiency remains relatively stable over time (19). ATM is crucial for lymphocyte development that relies on double-strand break repair, such as V(D)J and class-switch recombination of immunoglobulin genes.

In approximately 70% of classical A-T patients, ATM deficiency results in PID with highly variable features. Defective cell-mediated immunity (T-cell lymphopenia with reduced function of CD4+) is found in almost all patients, whereas deficiency in humoral immunity is more variable (decreased or absent of serum IgA, IgE, IgG2 and IgG4 levels, deficiency of specific antibody responses and gammopathy). The most frequent defect in the immunoglobulin profile is related to IgA and IgG2 deficiencies, which are diminished in 50% and 80% of cases, respectively (9). An estimated 10% of A-T patients have decreased IgA and IgG levels with normal to increased IgM levels, which is referred to as “hyper IgM phenotype” (3, 15, 19). In our cohort, we observed a mild to severe hypogammaglobulinemia, often low B and T cells but normal lymphocyte function. In line with the literature, we also noted a discrepancy between laboratory immunological abnormalities and the relative seldom increased vulnerability to systemic bacterial and severe viral or opportunistic infections, even before IVIG was started without antibiotic prophylaxis (20). Nevertheless, mild sino-pulmonary infections occur quite frequently and could be explained by the presence of swallowing difficulties, pharyngeal/thoracic muscle weakness and abnormal injury repair in a setting of immune dysregulation (3, 15, 19, 21). Lung disease is present in more than 70% of A-T patients, and 50% of them die in adolescence from respiratory failure in a context of irreversible parenchymal damage (emphysema, bronchiectasis and interstitial lung disease/pulmonary fibrosis) (3). The majority of patients with a mild A-T variant present themselves with normal immunological capacity (15). It was suggested that the residual ATM kinase activity mainly resulted in a less severe immunological phenotype than in patients without ATM kinase activity (8). Our three patients each carried a missense mutation that resulted in expression of some mutant ATM with some retaining kinase activity. The presence of decreased immunoglobulins with normal T/B cells, in the older patient 2, could be explained by a lower residual ATM kinase activity than in the others with the mild A-T variant, in a setting of age-associated immune deterioration. The subclinical antibody deficiency in young patients with the A-T variant might become clinically apparent with progressive aging of the immune system due to the lack of ATM protection (19). The titres of the vaccine-specific antibodies against tetanus, diphtheria, varicella, measles and pneumococcus were measured in 5 patients, except the one who had no B-cells (patient 1). All had high tetanus/diphtheria/measles-specific antibody levels. Varicella-specific antibody levels were negative in the younger patient; we therefore recommended him the Varicella vaccine (**Table 1**). Differences between the specific antibody levels against pneumococci could be explained by the different exposures to different pneumococcal serotypes and the time of vaccination in relation to the blood tests. Pneumococcal 13-valent conjugate vaccine (PVC 13) is recommended in these patients to reinforce protection against possible invasive pneumococcal diseases. A-T patients are expected to respond well to vaccination with decreases in specific antibody levels over time. Some expert guidelines recommend vaccination of all A-T patients with PVC 13 followed by the 23-valent pneumococcal polysaccharide vaccine every 5 years (15, 21). In A-T, the use of live vaccines is safe except for severely reduced T-cell numbers (CD4 < 500/mm^3^; CD8 < 300/mm^3^). Non-live vaccines are recommended in A-T as part of routine childhood vaccination programmes, except for patients receiving immunoglobulin replacement therapy. Annual vaccination with inactivated influenza vaccine and currently COVID-19 vaccines is recommended for all patients with A-T (15). Our mild and classical A-T patients, even those without B cells and very low T cells, tolerated well their MMR and varicella vaccine in infancy, showing a normal function of their cells.

Our study is a retrospective case series with only 6 patients, suggesting that A-T should not only be considered in the differential diagnosis of cerebellar ataxia beginning in childhood but also in children and adolescents with extrapyramidal movement disorders without other clinical features of A-T. Adults with unexplained movement disorders may also suffer from A-T (8). These patients with a milder A-T phenotype also have an almost normal immunological phenotype.

In conclusion, the path to diagnosis of A-T may be complex and delayed, as abnormal movements may be difficult to assess in a developing child. However, given the radiosensitivity, immunodeficiency and neurological impairment, early diagnosis is crucial for long-term life quality. The introduction of exome sequencing for unexplained neurological disorders may lead to the diagnosis of more cases of A-T and might further enlarge the spectrum of AT (22).

## Data Availability Statement

The datasets presented in this article are not readily available because it is a case series. Requests to access the datasets should be directed to Geraldine.BlanchardRohner@hcuge.ch.

## Ethics Statement

Ethical review and approval were not required for the study on human participants in accordance with the local legislation and institutional requirements. Written informed consent to participate in this study was provided by the participants’ legal guardian/next of kin.

## Author Contributions

GB-R and JF were involved in the conception of the paper. GB-R, AP, and LC wrote the first drafts of the manuscript. CK, JH, PB, FG-P, and PJ analyzed the cases. ER did the genetic analyses. HD and AT did the ATM activity signaling analyses. All authors contributed to the article and approved the submitted version.

## Conflict of Interest

The authors declare that the research was conducted in the absence of any commercial or financial relationships that could be construed as a potential conflict of interest.

## Publisher’s Note

All claims expressed in this article are solely those of the authors and do not necessarily represent those of their affiliated organizations, or those of the publisher, the editors and the reviewers. Any product that may be evaluated in this article, or claim that may be made by its manufacturer, is not guaranteed or endorsed by the publisher.
